# An Introduction to Practical Chemistry; Including Analysis

**Published:** 1857-01

**Authors:** 


*An Introduction to Practical Chemistry; including Analysis.* By JOHN E. BOWMAN, F.C.S.; Prof. of Practical Chemistry in King's College, London; Author of 'A Handbook of Chemistry,' &c. Second American, from the Second and Revised London Edition. Philadelphia, Blanchard & Lea, 1856.

This very valuable little work has been some time before the profession; and its merits are well known. To the student of practical chemistry it is one of the most convenient and useful volumes within his reach.

Copies of the three foregoing works were received through the well-known book establishment of D. B. Cooke & Co. of this city, where the works may be found by those wishing to purchase.

